# Preoperative physiotherapy education for patients undergoing colorectal cancer resection

**DOI:** 10.4102/safp.v65i1.5614

**Published:** 2023-01-06

**Authors:** Megan J. Whelan, Ronel Roos, Marelee Fourie, Heleen van Aswegen

**Affiliations:** 1 Department of Physiotherapy, Faculty of Health Sciences, University of the Witwatersrand, Johannesburg, South Africa

**Keywords:** preoperative education, framework, colorectal cancer, abdominal surgery, physiotherapy

## Abstract

**Background:**

Surgical resection is a common treatment for patients with colorectal cancer. Patients undergoing surgery are at risk of functional deterioration as a response to surgical stress. Furthermore, patients with cancer often present with systemic problems as well as a functional decline. The study aimed to create a framework for preoperative education for patients undergoing colorectal cancer resection.

**Methods:**

Five databases were utilised to find intervention-based studies describing the content, mode, setting and timing of delivery of preoperative education for patients undergoing abdominal surgery. Physiotherapists were purposively sampled to participate in a focus group session using a seven-step nominal group technique (NGT) with the goal to reach consensus on the proposed content of a preoperative patient education programme.

**Results:**

Seventeen studies were reviewed. Results indicate that the mode and timing of the education provided are heterogenous. Content included in the education programs described were breathing exercises, coughing techniques, verbal advice, physical exercises, surgical information, postoperative pain management, nutritional support, relaxation techniques and information about postoperative complications. Six physiotherapists participated in the focus group discussion. Ideas generated in the focus group were similar to those described in the literature.

**Conclusion:**

Results from both the narrative review and the focus group session assisted the authors to develop a framework for the content, timing, setting and mode of delivery of physiotherapy preoperative education for patients undergoing surgical resection for colorectal cancer.

**Contribution:**

The framework can be used to inform a physiotherapy preoperative education programme for patients undergoing surgery for colorectal cancer.

## Introduction

Colorectal cancer is the fourth most common type of cancer in South Africa.^1^ Treatment strategies for colorectal cancer are broad and include surgical resection, chemotherapy and radiation therapy.^2^ Patients with cancer often present with systemic problems as well as a functional decline because of the effects of the various treatments or as a result of cancer itself.^3^ Furthermore, patients undergoing major surgery are also at risk of functional deterioration because of impairment of muscular, cardiorespiratory and neurological function as a response to surgical stress.^4^ Emerging data from a cohort of patients from a private South African hospital have shown that surgical site infection and postoperative paralytic ileus were the most frequently reported postoperative complications in patients who underwent surgical resection for colorectal cancer.^5^

Prehabilitation has been defined as the process of improving an individual’s functional capacity to better withstand a stressful event such as surgery.^6^ There is research supporting the use of preoperative exercise strategies to optimise overall health and improve postoperative outcomes in patients undergoing surgery for various forms of abdominal cancers.^7^ In addition to this, psychological support and nutritional input have also been included to create a multimodal prehabilitation approach.^8^

Evidence suggests that educating patients on what to expect before surgery is beneficial by reducing postoperative anxiety, increasing patient satisfaction and improving postoperative outcomes such as length of stay in the hospital.^9^ Preoperative education is considered an essential part of the ‘Enhanced Recovery After Surgery’ (ERAS) recommendations.^10^ Effective management of patients with colorectal cancer requires input from various members of the multidisciplinary team. Surgeons and nurses usually provide preoperative education and counselling for this patient population.^11^ This is the current preoperative management of patients with colorectal cancer in a private hospital in South Africa. Typically, this comprises information about the nature of the surgical procedure, what to expect in the pre- and postoperative phases of their surgery, postoperative targets the patient is expected to meet (e.g. the instruction to exercise) and the proposed discharge date.^11^

The traditional ‘prehabilitation’ role of physiotherapists could be broadened to include educational input for patients undergoing surgery for colorectal cancer. Physiotherapy preoperative education is the process of providing targeted preparatory information to patients regarding their expected postoperative journey and associated participation in various exercises and activities of daily living.^12^

Current evidence supports the use of preoperative physiotherapy to reduce the risk of postoperative pulmonary complications in patients undergoing upper abdominal surgery.^13^ Preoperative physiotherapy interventions for patients in sub-Saharan Africa is a need that is largely unmet.^14^ The reason for this is unknown. Education (which can be considered prophylactic) is included in the scope of practice of South African physiotherapists, which is described in the *Health Professions Act 56 of 1974*.^15^ Before surgery, physiotherapists could assist patients with managing activity-related expectations after surgery, explaining postoperative physiotherapy treatment procedures, assisting with controlling preoperative lifestyle-related risk factors and providing other support as required by patients. If patients are only receiving postoperative physiotherapy and nothing during the vital preoperative period, one could surmise that they are missing out on an element of their care. The educational content that physiotherapists could deliver to patients undergoing surgery for colorectal cancer specifically is yet to be established.

This study aimed to create a framework for physiotherapy-specific preoperative education for patients undergoing abdominal surgery for colorectal cancer. Firstly, the framework was informed by a narrative review of published literature describing the mode of delivery, timing, setting and content of preoperative education material provided by physiotherapists to patients undergoing abdominal surgery. Secondly, a focus group conducted with experienced physiotherapists strengthened the content component of the framework as they reflected on essential components of such a program.

## Narrative review

A narrative review was performed to describe the content, timing, setting and mode of delivery of physiotherapy preoperative education for patients undergoing abdominal surgery for colorectal cancer described in the literature.

### Materials and methods

Two of the authors independently searched for articles to be included in the narrative review. Inclusion criteria were intervention studies (both randomised controlled clinical trials and quasi-experimental studies), papers published in English and studies investigating patients undergoing abdominal surgery who received preoperative education at any stage before their surgical procedure. Articles were excluded if patients were under the age of 18 years; patients did not undergo abdominal surgery; preoperative education was not included in the preoperative management; or if the authors felt that the preoperative education provided was not appropriate to be administered by a physiotherapist (i.e. outside of scope of practice).^15^ Papers that were narrative reviews, scoping reviews or systematic reviews on the topic were excluded. Because of the wide range of information required for patients undergoing abdominal surgery, we decided not to exclude articles where the study participants underwent abdominal surgery for causes other than colorectal cancer if it was still related to preoperative education.

Search terms included ‘preoperative education’, ‘physiotherapy’, ‘abdominal surgery’ and ‘cancer’. The databases utilised for the search included Cumulative Index to Nursing and Allied Health Literature (CINAHL), ScienceDirect, Google Scholar, PubMed and Physiotherapy Evidence Database (PEDro). Duplicate articles were removed. Both authors independently reviewed the content of the articles using a simple data extraction table. The reference lists of the articles included were manually searched for more articles to be included in the review. Data were extracted from the studies meeting the inclusion criteria. Information extracted included: author, year of publication, country of origin, aim or purpose, study sample, methodology, intervention type or mode, intervention content, duration of intervention, measurement of outcomes and key findings (Appendix 1). The first author proceeded to use the Joanna Briggs Institute Critical Appraisal Checklists for randomised controlled trials and quasi-experimental studies.

### Data analysis

For the narrative review, pertinent information for each paper considered for inclusion was extracted by two authors and recorded on study-specific data extraction forms.

### Results

Seventeen papers were included in the narrative review. A flow diagram describing the process of article selection for the review is displayed in Figure 1. Table 1 summarises the type of study, patient population, timing and mode of delivery, content, language and setting of preoperative education delivered to the patients.

**FIGURE 1:**
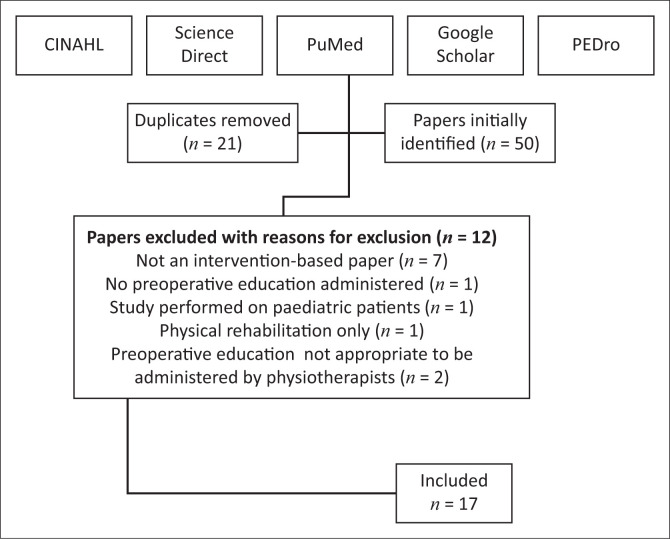
Flow diagram of article selection.

**TABLE 1:** Synopsis of studies included in the narrative review on preoperative patient education.

Paper	Type of study	Patient population	Timing	Mode	Setting for education	Language	Content of education
Dronkers et al.^19^	Single-blind pilot randomised controlled trial	Elective abdominal oncological surgery	2–4 weeks preoperatively	Face-to-face counselling	Outpatient clinic	Not specified	Home-based exercise advice; importance of physical conditioning; breathing exercises; coughing
Li et al.^24^	Prospective pre- and postintervention study	Elective colorectal cancer resection	Variable depending on surgery wait time	Face-to-face counselling, digital	Outpatient clinic	English, French	Nutrition and modifiable dietary behaviour advice; anxiety reduction; home-based exercise advice (aerobic and resistance)
Gillis et al.^7^	Parallel-arm single-blind superiority randomised controlled trial	Colorectal cancer resection	Approximately 6 weeks preoperatively	Face-to-face counselling, written or pictorial	Outpatient clinic	Not specified	Home-based exercise advice (aerobic and resistance); nutrition advice; anxiety reduction
Samnani et al.^28^	Randomised control trial	Abdominal surgery	Not specified	Face-to-face counselling	Outpatient clinic, hospital emergency department	English, Urdu	Surgical information; early mobilisation information (intervention group only)
Boden et al.^29^	Prospective, pragmatic, multicentre, patient- and assessor-blinded, parallel group, randomised placebo-controlled superiority trial	Major upper abdominal surgery	Within 6 weeks preoperatively	Written or pictorial, face-to-face counselling (intervention group only)	Outpatient clinic	English	Postoperative pulmonary complications; breathing exercises; postoperative ambulation information
Rodrigues et al.^21^	Prospective, interventional, single-centre controlled clinical trial	Abdominoplasty	One week before surgery	Face-to-face counselling, written or pictorial	Outpatient clinic	Not specified	Breathing exercises
Howard et al.^23^	Propensity-matched retrospective chart review	Major abdominal and thoracic surgery	At least 2 weeks preoperatively	Digital, written or pictorial	Outpatient clinic	Not specified	Walking information; breathing exercises; nutrition; stress management; smoking cessation
Beck et al.^30^	Convergent design	Elective abdominal oncological surgery (ovarian and colorectal cancer)	Not specified	Written or pictorial	Outpatient clinic	Not specified	Exercise, nutrition, relaxation techniques, smoking cessation, alcohol cessation; other preparation
Raj and Kathyayani^26^	Pre-experimental one group pretest–post-test design	Elective abdominal surgery	Not specified	Face-to-face counselling	Not specified	Kannada	Breathing exercises
Al-Reda and Rajha^27^	Quantitative quasi-experimental, pre-test and post-test design.	Abdominal surgery	More than 1 day preoperatively	Face-to-face counselling	Not specified	Arabic	Breathing exercises
Berthelsen et al.^25^	Randomised single-blinded controlled trial	Abdominal surgery	One to several weeks preoperatively	Face-to-face counselling	Outpatient	Swedish	Early ambulation; breathing exercises; physical activity and activity restrictions after discharge
Tejedor et al.^20^	Retrospective nonrandomised cohort study	Elective colorectal cancer resection	Not specified	Not specified	Not specified	Not specified	Surgical information; nutrition
Ünver, Kıvanç and Alptekin^16^	Descriptive cross-sectional study	Abdominal surgery	Not specified	Not specified	Hospital ward	Not specified	Breathing exercises
Tripathi and Sharma^41^	Quasi-experimental pilot study	Abdominal surgery	Not specified	Face-to-face counselling, written or pictorial	Not specified	Not specified	Breathing exercises
Klaiber et al.^18^	Cluster randomised controlled trial	Major elective visceral surgery	One day before surgery	Group seminar, written or pictorial	Inpatient	Not specified	Postoperative complication prevention; acute pain therapy and coping strategies; postoperative ambulation; wound support
Boden et al.^22^	Nested mixed-methods randomised-controlled study	Upper abdominal surgery	Within 6 weeks preoperatively	Written or pictorial, face-to-face counselling (intervention group only)	Outpatient clinic	English	Postoperative pulmonary complications; breathing exercises; postoperative ambulation information
Boden et al.^30^	Multicentre randomised controlled trial	Upper abdominal surgery	Within 6 weeks preoperatively	Written or pictorial, face-to-face counselling (intervention group only)	Outpatient clinic	English	Postoperative pulmonary complications; breathing exercises; postoperative ambulation information

### Health professionals involved in the delivery of preoperative education

There are data to suggest that nurses and nursing students are often responsible for the delivery of preoperative education on breathing exercises to patients before abdominal surgery.^16,17,18^ In some cases, physiotherapists are responsible for the delivery of preoperative education to patients undergoing abdominal surgery;^19,20,21^ however, in most studies, various members of the multidisciplinary team, including physiotherapists, nurses, physicians, surgeons, dietitians and psychologists, contribute to the content of preoperative education provided because of the wide variety of information required.^7,13,22,23,24,25^

### The outcome of preoperative education

Because many studies utilised preoperative education as part of a larger prehabilitation program, one often cannot determine the impact of preoperative education alone. However, studies by Ünver, Kıvanç and Alptekin,^16^ Boden (2017, 2018, 2020)^12,22,30^, Raj and Kathyayani,^26^ Al-Reda and Rajha,^27^ Beck et al. (2020)^31^ and Samnani et al.,^28^ which employed preoperative education as the primary intervention, can be used to discuss the impact of such education on postoperative outcomes for patients who had abdominal surgery.

One study showed that preoperative education in the form of 30 min of face-to-face interaction and an instructional booklet reduced the incidence of postoperative pulmonary complications.^29^ Their findings suggest that preoperative education is likely to be a cost-saving strategy.^30^ Similarly, another study showed that there was only a 4% incidence of postoperative complications where preoperative teaching of breathing exercises was used for patients undergoing elective abdominal surgery.^26^ Furthermore, a third study showed that patients who were exposed to an educational breathing exercise program before their abdominal surgery showed greater improvements in postoperative lung function tests compared with patients who received standard preoperative care.^27^ In contrast, Klaiber et al.^18^ found a similar incidence of postoperative complications in patients who received an informative brochure compared with those who attended a training seminar and received the brochure. There were no significant differences between the groups regarding pain perception, anxiety, quality of life or depression.^18^

### Recall and/or revision of information provided

One study found that face-to-face education is highly memorable and has high treatment fidelity for patients undergoing upper abdominal surgery.^22^ Another study found a strong correlation between those patients who actually received the education on breathing exercises and those patients who performed the exercises postoperatively.^16^ Berthelsen et al. found that patients recalled no more than 27% of the preoperative information that was provided to them when asked questions a few days later. There were small, nonsignificant differences between the control group (who received written information) and the experimental group (who received the information utilising the teach-back method.^25^ Results of another study showed that patients prepared for cancer-related abdominal surgery in various ways, which were not limited to the written recommendations provided by the multidisciplinary team.^31^

## Focus group discussion

Various methods are used in research for problem-solving and idea generation.^32^ The nominal group technique (NGT) is a form of consensus-seeking designed to achieve general agreement around a particular topic.^33^ The benefits of an NGT focus group are that all participants are allowed to voice their opinions, and the results are obtained quickly.^33^

### Participant information and inclusion criteria

Physiotherapists known to the authors and working in surgical intensive care and high-care units in Johannesburg, South Africa, were purposively sampled to participate in a focus group session using the NGT. Physiotherapists were invited to participate in our study if they had at least five years of experience working in acute care or a surgical ward setting with patients who had abdominal surgery for various conditions or those specifically working in a surgical or oncology setting. Twelve physiotherapy clinicians from 10 different hospitals in both the private and public sectors, who were working in the Gauteng province and met the inclusion criteria, were invited to participate. Furthermore, two patients known to the authors who had undergone surgical colorectal cancer resection were invited to join the session.

### Procedure

Eligible participants were invited to attend a focus group session at the Physiotherapy Department at the University of the Witwatersrand in Johannesburg in October 2019. Participants completed a demographic questionnaire before the start of the session. The one session was audio-recorded with written informed consent from all participants. A transcription of the recording was not made and therefore not verified by participants. The identity of all participants is stored in a secure password-protected folder to which only the main author has access. A seven-step NGT was used to answer the following stimulus question: ‘What information should be included in preoperative physiotherapy education for patients undergoing abdominal surgery for colorectal cancer to minimise the development of postoperative complications?’ The session was facilitated by the authors, one of whom was experienced in NGT methodology. One author led the session and the other took computerised notes throughout the session. The author who led the session was responsible for inviting the participants, analysing the results and communicating with participants after the session to finalise consensus on the final ranking.

Participants were asked to formulate ideas according to ‘respiratory system considerations’, ‘wound considerations’, ‘cancer-related considerations’ and ‘other considerations’. Participants were given time to formulate their individual ideas and write each idea onto a coloured self-adhesive paper. Ideas were then shared among the group where clinicians were invited to paste their ideas on the wall for everyone to see. Following this, clarification and clustering of ideas ensued through a group discussion where similar ideas were grouped and duplicate ideas were discarded. Participants were then asked to rank their ideas in order of importance on individual ranking sheets. General ranking of ideas was done rather than ranking for ‘respiratory system considerations’, ‘wound considerations’, ‘cancer-related considerations’ and ‘other considerations’ specifically. Participants were asked to rank the categories of ideas according to the 10 they felt to be most important. During the session, notations were kept on a laptop to ensure that the ideas shared were not lost. Notes and ideas were captured in a Microsoft Excel spreadsheet (Microsoft Corporation, Redmond, Washington, United States). After completion of the session, content from the self-adhesive papers was compared with the information captured in the Excel spreadsheet to ensure that all findings aligned. Results of the category rankings were tallied by the author who led the session. Categories that received the most votes were ranked first, and those that received the least were ranked last. The final collective ranking of ideas was determined and sent to each participant via e-mail after the session. Participants were given one week to give feedback on the final ranking to achieve overall consensus.^34^ One hundred percent consensus was considered achieved if all six participants agreed (partially or fully) with the final ranking tally. Table 2 displays a summary of the NGT methodology used in the focus group session.

**TABLE 2:** Summary of the nominal group technique methodology used in the focus group session.

Step	Duration	Description
Step 1: Stimulus question posed	10 min	Following the introduction, a detailed explanation of the methodology and expectations was given; written consent was obtained; and the stimulus question was posed by the researcher to the participants.
Step 2: Brainstorming	15 min	The participants were asked to individually formulate ideas according to ‘respiratory considerations’, ‘wound considerations’, ‘cancer considerations’ and ‘other considerations’. Each participant documented their ideas on separate colour-coded self-adhesive notes.
Step 3: Sharing of ideas	45 min	Ideas were then verbally shared among the group, and each idea was stuck on the wall in the various categories for all participants to observe. Each participant was given a chance to share one idea at a time until all the ideas had been shared.
Step 4: Clarification and clustering	65 min	A group discussion ensued, led by the researcher. The researcher numbered and read each idea aloud, and participants guided the placement of the ideas on the wall. Similar ideas were clustered together, and duplicate ideas were discarded. Participants were invited to interrogate any ideas that were displayed, as well as question how the clusters of ideas were being developed. During this process, there was no onus on the participant whose idea was under discussion to take ownership of the idea.
Step 5: Ranking of ideas	20 min	A final list of ideas for each category was displayed on the wall. A ranking list was provided for each participant. Each participant was asked to rank the ideas in terms of their importance across all categories. This step was completed individually by all participants in silence. Participants handed in their ranking sheets to the researcher at the end of the session.
Step 6: Final tally	24 h	Votes were captured in a self-designed Excel database. The ideas were ranked based on the votes received. These results were e-mailed to participants within 24 h of the focus group meeting.
Step 7: Final ranking and consensus	1 week	The participants were invited to give feedback on the final ranking to achieve overall consensus on the ideas generated around the stimulus question posed.

#### Data analysis

Statistical analysis for the focus group was performed using the Statistical Package for the Social Sciences (SPSS) version 25 (IBM Corporation, Armonk, New York, United States). Descriptive statistics were used to summarise the data. Demographic data were analysed using mean (standard deviation [s.d.]) or numbers (percentages).

#### Results

### Demographics

Twelve physiotherapists were invited to participate in the focus group. Six physiotherapists responded to the invitation and agreed to participate in the session. The remaining six did not respond to the invitation; the reason for this was unknown. Two patients were invited: one could not attend because of prior commitments and the second had initially agreed to participate but was unable to attend on the day because of a family emergency. The mean age of participants was 36 years (s.d. = 8.5). There were five female (83%) and one male participants. One participant reported having a master’s degree in cardiopulmonary physiotherapy, four had undergraduate physiotherapy degrees and one had an undergraduate degree with a postgraduate diploma in cardiopulmonary rehabilitation. Two participants reported having more than 10 years of overall experience working in acute care or a surgical ward setting, and the remaining four (67%) reported having between 5 and 10 years of experience. All six participants worked in the private sector. Table 3 shows the physiotherapy areas of interest of the participants. Three participants reported having more than one area of interest.

**TABLE 3:** Areas of interest expressed by participants of the focus group session.

Area of interest	Percentage
Cardiopulmonary	46
Pain	15
Women’s health	23
Oncology	8
Neurology	8

#### Focus group results

**FIGURE 2:**
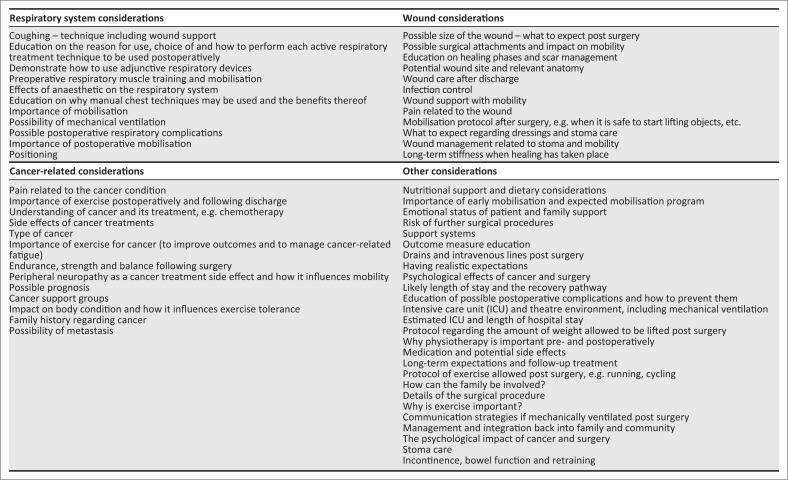
Ideas suggested by focus group participants related to the ‘respiratory system considerations’, ‘wound considerations’, ‘cancer-related considerations’ and ‘other considerations’ domains.

There were 108 ideas generated by the participants across the four domains. Twenty-eight percent of the ideas (*n* = 30) were generated in the ‘respiratory system considerations’ domain, 21% (*n* = 23) in the ‘wound considerations’ domain, 15% (*n* = 16) in the ‘cancer-related considerations’ domain and 36% (*n* = 39) in the ‘other considerations’ domain. Figure 2 displays the ideas suggested in each of the domains once duplicates were removed, and some ideas were combined as they were found in more than one domain. Following the group discussion where ideas were clustered together, 17 clusters of ideas remained. Concerns were raised about excluding certain categories that led to the ranking of all 17 categories instead of just 10. Verbal consensus was reached by all six participants during the session to include all 17 categories in the final ranking. Each individual then voted on the final ranking of the 17 categories. None of the participants had the same individual ranking of the 17 categories. A final combined ranking list was developed as described in the methodology and e-mailed to participants after the session was concluded. All participants confirmed via e-mail that they agreed with the final ranking results. The authors considered this to confirm 100% consensus with the final ranking. Table 4 summarises the categories in order of importance following the final ranking tally.

**TABLE 4:** Final ranking of categories for inclusion in preoperative education for patients with colorectal cancer.

No.	Description of category
1	Education on active breathing techniques and why they are important
2	Education on the development of possible respiratory complications
3	Wound support with activity – how and why?
4	Positioning to optimise respiratory function
5	Education on how to perform active breathing techniques
6	Pain – cancer, wound, medication
7	Importance of mobilisation – education, complications and outcome measures
8	Passive physiotherapy techniques to assist with sputum clearance
9	Medical and surgical journey and surgical expectations
10	Wound care and infection control
11	Scar management during the phases of healing
12	The rehabilitation protocol – short-term, long-term, including post discharge (pathway of care)
13	Side effects of chemotherapy and radiation
14	Importance of the multidisciplinary team (support, family, dietician, stoma nurse, etc.)
15	Cancer – types, prognosis and outcomes
16	Exercise and cancer
17	Other complications – bladder and bowel

**FIGURE 3:**
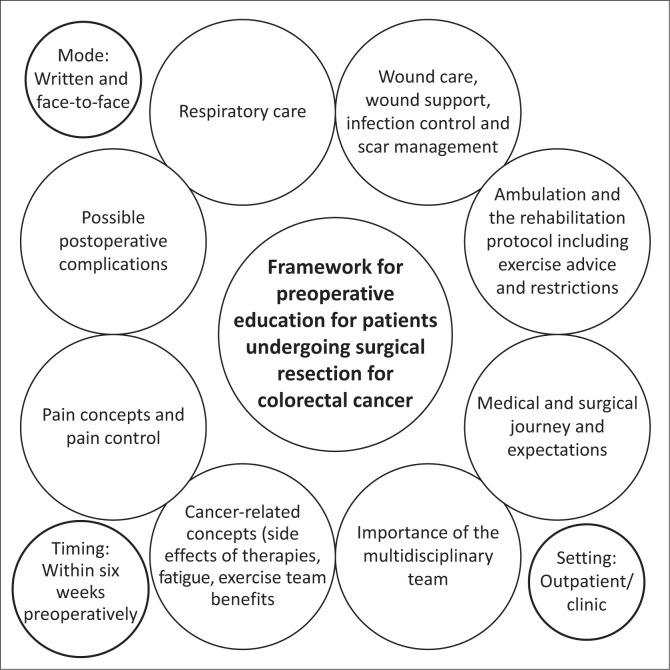
Framework for preoperative education for patients undergoing surgical resection for colorectal cancer.

The first author used the summary of the narrative review and results of the focus group session to develop a combined framework for physiotherapy preoperative education. The other two authors reviewed the framework developed and gave feedback, following which changes were made as necessary (Figure 3).

## Discussion

Various studies were identified that utilised preoperative education strategies for patients undergoing abdominal surgery. The timing of preoperative education delivery described in the literature appears to be variable. Preoperative education is provided by various members of the multidisciplinary team.^7,13,22,23,24,25^ There is evidence that preoperative physiotherapy education prior to abdominal surgery has shown high treatment fidelity, is highly memorable, leads to lower hospital costs and reduces postoperative pulmonary complications.^22,26^ However, it appears that physiotherapists are often underutilised when it comes to the provision of preoperative education for patients undergoing abdominal surgery. For example, the provision of education regarding breathing exercises falls within the scope of physiotherapy practice,^15^ although currently breathing exercise education is being provided by nurses in multiple settings.^16,26^ Although nurses are able to provide general advice regarding breathing exercises, physiotherapists would educate patients in more detail.^15,35^

Content included in the education programs included breathing exercises, coughing, verbal advice on trunk and pelvic mobility exercises and leg mobility exercises, surgical information, postoperative pain management, nutritional support, relaxation techniques and information about postoperative pulmonary complications and their prevention with early postoperative ambulation. The question remains whether the content of preoperative educational information provided to patients undergoing abdominal surgery by physiotherapists could be expanded upon to include other concepts and whether this could have any impact on their postoperative outcomes.

Many of the ideas presented in the final NGT ranking by the participating physiotherapists were similar to those described in the education program provided to patients in the studies by Boden et al.^13,30^ Our results had more information related to cancer, which is appropriate given that the clinicians were tasked with determining the information that should be included in preoperative physiotherapy education for patients undergoing abdominal surgery for colorectal cancer specifically. Categories such as treatment side effects (chemotherapy and radiation) and cancer (type, prognosis, etc.) were ranked outside the top 10 items in our study. This could be because some of the participants verbalised during the session that although these concepts are important, discussion of these matters could be outside the scope of physiotherapy practice.

Based on the opinion of the clinicians who attended the focus group, concepts such as the postoperative rehabilitation journey and physiotherapy management of incontinence could also be included in a physiotherapy preoperative education program. For patients undergoing surgery for colorectal cancer specifically, preoperative education on cancer-related fatigue and the associated benefits of exercise and the impact of lifestyle-related risk factors on postoperative outcomes could be beneficial for patients undergoing abdominal surgery for the tumour resection.

Results of a systematic review and meta-analysis showed that multimodal exercise interventions are effective in the management of cancer-related fatigue.^36^ Preoperative functional performance levels and physical activity are predictors of acute postoperative outcomes in South African patients undergoing colorectal cancer resection.^5^ This supports the decision to include physical activity and information regarding other lifestyle-related factors to effect changes in acute and long-term outcomes in this patient group.

‘Early Recovery After Surgery’ protocols are multidimensional and consider aspects of preoperative, perioperative and postoperative care aimed at enhancing recovery following surgery.^10^ The ERAS pathway helps reduce the incidence of postoperative complications and hospital length of stay in patients undergoing major open abdominal surgery for colorectal cancer. Because of the fact that ERAS guidelines encourage a multidisciplinary approach, it is important that patients are aware of the other members of their care team. Over the past few years, there has been a rise in multidisciplinary management of patients with cancer. An important component of ERAS principles is postoperative analgesia.^10^ Although the optimal modality for postoperative analgesia control is still up for debate, poorly controlled analgesia is associated with impaired mobility and poor postoperative outcomes.^10^ Physiotherapists are able to advocate for their patients to receive appropriate pain control, which has the potential to improve acute postoperative outcomes such as hospital length of stay. Another component of the ERAS pathway is preoperative counselling where patients are given information about their medical and surgical journey, which would support the addition of this component to our framework.^10^

Evidence supporting the effectiveness of breathing exercises to prevent postoperative pulmonary complications is inconclusive. On the one hand, results of a recent randomised controlled trial found that the active cycle of breathing technique is more effective than autogenic drainage in improving thoracic expansion, peak expiratory flow rate and inspiratory capacity in patients who underwent abdominal surgery.^37^ Alternatively, results of a cluster randomised controlled trial showed that the addition of deep breathing exercises to physiotherapy-directed early mobilisation following abdominal surgery had no impact on the incidence of postoperative pulmonary complications when compared with early ambulation in isolation.^38^ However, given the fact that active breathing techniques were at the top of the final ranking after the NGT, and were included in several of the studies reviewed, the authors retained it as part of ‘respiratory care’ in the framework.

Early ambulation after surgery is one of the components of the ERAS guidelines.^10^ Browning et al.^39^ performed a study to determine how much uptime is performed in the first four days following upper abdominal surgery. Results showed that the quantity of upright mobilisation is low in these patients.^39^ Less uptime was a predictor of longer hospital length of stay.^39^ Similarly, results from a more recent prospective cohort study in older patients who underwent emergency abdominal surgery found that delayed mobilisation was associated with poor postoperative outcomes.^40^ This highlights the need to educate patients on the benefits of early upright positioning and mobilisation following surgery.

The time period between diagnosis and commencement of treatment in South African patients with colorectal cancer remains heterogenous. However, if referred timeously to physiotherapists, many patients could benefit from preoperative interventions such as physiotherapy education. Doctors and other primary care practitioners could play a vital role in the referral of patients for various rehabilitation services at the time of diagnosis.

### Limitations

Although 17 studies were identified and included in the narrative review, only eight were randomised controlled trials.^7,18,19,22,25,28,29,30^ The remaining nine studies utilised a variety of other interventional study designs. Because of the varying strengths of the designs of research papers included, it is difficult to accurately draw solid conclusions for this review. Rather, the information gathered regarding content, timing and method of delivery of preoperative education can be used to guide future studies.

Because the clinicians were purposively sampled to participate in the focus group session, selection bias cannot be ruled out. Furthermore, because the focus group session was facilitated by the authors, there was also a risk of bias during the session that cannot be ignored. There were a small number of participants who took part in the NGT; however, the sample still fell within the recommended sample size for an NGT session.^33^ Our study only included physiotherapy clinicians who work in the private sector, despite the invitation being extended to clinicians working in the public sector. Clinicians working in the public sector may have presented different ideas during the NGT session. Furthermore, although two patients were invited to participate in the session, we only had physiotherapy clinicians in attendance.

The study populations described in the narrative review were heterogenous and not limited to patients with colorectal cancer. However, because the framework was also based on the results of the focus group, the framework presented could be considered useful for patients with colorectal cancer. However, further validation and testing will be required before this can be used in clinical practice. The framework developed was not reviewed by members of the focus group, and consensus was only achieved by the authors of this paper.

## Conclusion

Preoperative physiotherapy education reduces the incidence of postoperative pulmonary complications, is memorable and has high treatment fidelity for patients undergoing abdominal surgery. Several authors investigated the use of physiotherapy preoperative education for patients undergoing abdominal surgery. Various modes of delivery are described, and the timing of education interventions is heterogenous and case dependent. The preliminary framework developed for preoperative physiotherapy education delivery to patients undergoing surgical resection for colorectal cancer provides the content, timing, setting and mode of delivery. Further research is needed to investigate the impact of a comprehensive physiotherapy preoperative education program on postoperative outcomes in South African patients undergoing colorectal cancer resection.

## Figures and Tables

**FIGURE 1-A1:**
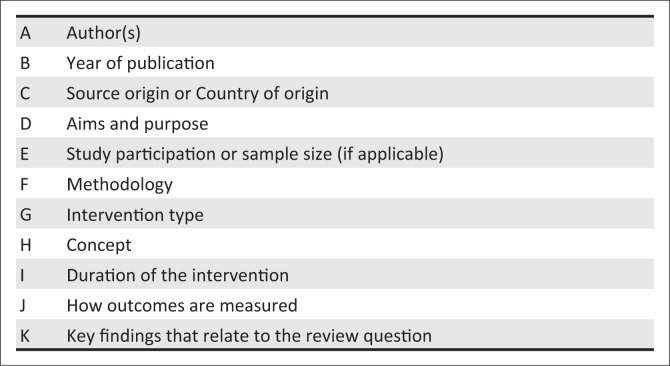
Extraction form for narrative review.
